# Associations Among Anxiety, Sleep Quality, and Binge Eating in Hispanic and African American/Black Early Adolescents

**DOI:** 10.3390/children13060761

**Published:** 2026-05-30

**Authors:** Norma Olvera, Molly R. Matthews-Ewald, Tamal J. Roy, Rhonda Scherer, Luz M. Garcini, Consuelo Arbona

**Affiliations:** 1 Department of Psychological, Health & Learning Sciences, University of Houston, 3623 Cullen Blvd., McElhinney Hall, Room 3001, Houston, TX 77204, USA; tjroy@cougarnet.uh.edu (T.J.R.); rlschere@central.uh.edu (R.S.); consueloa@central.uh.edu (C.A.); 2 WhitworthKee Consulting, LLC, Washington, DC 20003, USA; mmatthewsewald@whitworthkee.com; 3 Department of Psychological Sciences, Kinder Institute for Urban Research, Rice University, 6100 Main St., Kraft Hall, Houston, TX 77005, USA; lmg7@rice.edu

**Keywords:** binge eating, Hispanic, African American, black, anxiety, sleep quality

## Abstract

**Highlights:**

**What are the main findings?**

- Higher levels of binge eating were significantly associated with higher anxiety scores among early adolescents, independent of gender.
- Higher levels of binge eating were significantly associated with poorer sleep quality among early adolescents, with this relationship remaining consistent across genders.

**What are the implications of the main findings?**

- Binge eating prevention efforts should target Hispanic and African American/Black boys and girls during early adolescence.
- Interventions targeting binge eating in Hispanic and African American/Black youth should incorporate screening and treatment for anxiety and sleep quality.

**Abstract:**

**Background/Objectives**: Binge eating behavior has been deemed a significant factor associated with adolescent obesity. Understanding the underlying factors contributing to binge eating is critical, particularly among youth at risk of obesity. Hispanic and African American/Black children and adolescents experience disproportionately higher rates of obesity compared to their White peers. This study investigated the associations among anxiety, sleep quality, and binge eating among 315 Hispanic and African American/Black boys and girls, while controlling for demographic and adiposity covariates. **Methods**: Participants completed self-report surveys and underwent objective assessments of height, weight, and body fat percentage. **Results**: Descriptive analyses indicated a mean age of 10.71 years for boys and 10.92 years for girls. Most participants (71.9%) were classified as overweight or obese, and 94.1% were U.S.-born. Hierarchical regression analyses showed that both anxiety (*β* = 0.24, *p* < 0.001) and sleep quality (*β* = 0.23, *p* < 0.001) were significantly linked with higher levels of reported binge eating, after controlling for age, ethnicity, gender, and body fat percentage. The full model accounted for 15.4% of the variance in binge eating. **Conclusions**: These findings highlight the importance of targeting anxiety and sleep quality in interventions aimed at reducing binge eating among Hispanic and African American/Black early adolescents.

## 1. Introduction

Despite attention and concerted prevention and intervention efforts, childhood obesity remains a major public health issue in the United States (U.S.), disproportionately affecting racially and ethnically minoritized youth. Data from the National Health and Nutrition Examination Survey (NHANES) 2017–2020 indicate that obesity prevalence among children and adolescents ages 2–19 years was approximately 26% among Hispanic youth and 25% among non-Hispanic Black youth, compared with 17% among non-Hispanic White youth [1,2]. Further, children and adolescents with excess weight are more likely to engage in disordered eating behaviors or receive an eating disorder diagnosis than their normal-weight peers [3,4,5]. For example, obesity has been linked to binge eating and binge-eating disorder (BED). According to the American Psychiatric Association [6], BED is characterized by recurrent episodes of consuming unusually large amounts of food within a short period of time, accompanied by a sense of loss of control and significant psychological distress. Although clinical BED is relatively uncommon in children and adolescents, subthreshold binge eating behaviors, such as out-of-control eating, are considerably more prevalent in youth [7]. Longitudinal research suggests that children who exhibit higher levels of overeating are more likely to engage in binge eating behaviors during adolescence, which can subsequently lead to a clinical BED diagnosis [8]. During adolescence, youth experience a period of increased body awareness and sociocultural pressures, which may contribute to gender differences in binge eating behaviors. A desire to meet thin ideal standards and body dissatisfaction may be associated with loss-of-control eating and emotional eating behaviors among adolescent girls [9]. In contrast, boys may be more likely to engage in disordered eating driven by muscularity-oriented ideals, which may include overeating or binge-type behaviors in pursuit of increased muscle mass rather than weight loss [10].

Psychosocial influences are also important in understanding gender differences in binge-eating behaviors. Females are more likely to report engaging in binge eating in response to negative emotions such as stress and depressive symptoms, consistent with affect-regulation models of disordered eating [11]. In contrast, males are less likely to report these behaviors, potentially reflecting broader patterns of under-recognition of eating disorders among males [12]. This underreporting is associated with lower rates of help-seeking, which may contribute to underdiagnosis and delays in intervention [13]. Emerging evidence further indicates that eating disorders in males are frequently under-recognized and under-treated, potentially resulting in greater illness severity and poorer long-term clinical outcomes [14].

Although binge eating is present in all racial and ethnic groups in the U.S., disparities exist in prevalence, diagnosis, and treatment, all of which warrant attention. Historically, research has generally shown higher rates of binge eating among White youth populations. However, more recent epidemiological evidence suggests that Hispanic and African American youth and adult populations may exhibit comparable or even higher rates of binge eating behaviors and BED relative to their White counterparts [15,16,17]. Moreover, individuals from racially and ethnically underserved groups are less likely to receive a diagnosis or treatment, contributing to under-recognition of eating disorders, including BED, in these populations [18]. Sociocultural factors such as acculturation and obesity may contribute to binge eating behaviors among Hispanic populations in the United States. Greater acculturation to U.S. culture has been associated with higher body mass index and increased obesity risk among Hispanic individuals, suggesting that sociocultural environments may influence weight-related health outcomes and potentially eating behaviors [19]. Additionally, Hispanic adolescents with overweight or obesity are more likely to engage in unhealthy weight-control practices and disordered eating behaviors, including binge eating, compared with their normal-weight peers [15].

Likewise, sociocultural and environmental factors, including socioeconomic conditions, body weight status, and psychosocial stressors, may contribute to binge-eating behaviors among African American youth. African American children experience higher rates of overweight and obesity compared with their non-Hispanic White peers, which may increase vulnerability to disordered eating behaviors such as binge eating [20]. African American adolescents with overweight or obesity are also more likely to report disordered eating behaviors compared with their normal-weight peers [21]. Additionally, experiences of racial or ethnic discrimination have been linked to a significantly increased risk of BED. Compared with their peers, adolescents who reported experiencing discrimination had approximately three times higher odds of having BED [22]. Together, these findings suggest a potential role of sociocultural and environmental factors in binge-eating risk among racially and ethnically diverse youth.

A variety of theoretical perspectives, including affect regulation, psychosocial stress, and restraint theory, offer valuable insight into how binge eating behaviors develop [23,24,25]. These frameworks support the etiology of stressors triggering negative emotions, which some individuals attempt to alleviate through binge eating. Restraint theory further posits that dietary restriction, and dysregulated eating patterns can heighten psychological deprivation, thereby strengthening cravings and weakening self-regulation [26]. These dynamics are particularly relevant during adolescence, a developmental stage characterized by increased focus on body image and ongoing development of self-control skills [27]. Persistent engagement in binge eating as a response to distress can reinforce excessive calorie consumption and lead to weight gain, which may contribute to a cyclical relationship between binge eating, weight gain, and emotional distress [28]. These theories, combined with the extant literature, suggest that youth who are overweight or have obesity may experience increased vulnerability to binge eating behavior, likely due to heightened stress, frequent experiences of stigma or teasing related to weight, and potential disturbances in physiological appetite regulation linked to excess body fat [29,30].

Research has increasingly demonstrated the relationship between anxiety, sleep disturbances, and disordered eating. For instance, anxiety and emotional dysregulation have been consistently linked to binge eating [31,32], whereas sleep disturbances have been associated with an increased risk of binge eating behaviors and BED [33,34]. Emerging evidence suggests that anxiety and sleep quality may interact to influence emotional eating in Hispanic boys and girls [35]. Elevated stress levels may also contribute to increased anxiety and difficulties with emotional regulation, which have been associated with binge-eating behaviors [31,32]. Increased anxiety has been associated with difficulties in both sleep onset and sleep maintenance, contributing to poorer sleep quality and reduced sleep duration [36]. Sleep disturbances, including insomnia and shorter sleep duration, are also commonly observed among individuals with BED, which may exacerbate loss-of-control eating, potentially through effects on reward processing and impulse regulation in the brain [33]. Evidence from the Adolescent Brain Cognitive Development Study (*N* = 9428; mean age = 12 years; 49% female; 44% non-White) indicated that adolescents with clinically significant sleep disturbance had 262% higher odds of developing BED between years two and three of the study. Sleeping fewer than nine hours per night has been associated with a higher risk of binge-eating behaviors among adolescents [34]. Sleep disturbances can disrupt appetite-regulating hormones, including increased ghrelin and decreased leptin levels, which have been associated with increased hunger and food intake [37,38]. Collectively, these findings suggest that stress, anxiety, and sleep disturbances may play a significant role in the development and maintenance of binge eating through both biological and behavioral processes.

Despite growing research on binge-eating behaviors among adolescents, several gaps remain in the literature. Most of the existing studies have focused on adult populations, with few studies examining factors associated with binge eating during early adolescence [39]. Although ethnic/racial disparities in obesity and binge-eating risk have been documented, limited research has examined the relative contribution of anxiety and sleep disturbances to binge-eating behaviors among ethnic minority youth, and to what extent gender may moderate these associations. Therefore, this study addressed the following research questions: (1) What is the contribution of anxiety and sleep quality to early adolescents’ reports of binge eating in Hispanic and African American/Black boys and girls after controlling for age, gender, ethnicity, and body fat percentage? (2) To what extent does gender moderate the relationship between anxiety and sleep quality to binge eating behavior among Hispanic and African American/Black adolescents? Based on the extant literature, the following hypotheses are proposed: (1) Higher anxiety scores and poorer sleep quality will be associated with increased binge eating; and (2) The relationship among anxiety, sleep quality, and binge eating will be moderated by gender, with girls exhibiting stronger relationships than boys.

## 2. Materials and Methods

### 2.1. Participants

This study used baseline data from 371 Hispanic and African American/Black early adolescents. A total of 56 cases were removed due to missing data on key variables, resulting in a final analytic sample of 315 participants. Data were drawn from different cohorts of participants who enrolled in a 4-week wellness summer program from 2016–2019 and a 12-week mindful afterschool program from 2017–2019. The eligibility criteria for participants included being (1) self-identified as Hispanic or African American/Black; (2) between the ages of 9 and 14 years; (3) at risk for overweight or having overweight (body mass index [BMI] > 85th < 95th percentile) or obesity (BMI > 95th percentile); (4) free of any physical disability that would prevent them from fully participating in the exercise component of the programs; and (5) able to participate for the entire duration of the selected program. Participants were recruited via printed flyers, media announcements, and referrals from physicians and school nurses in a metropolitan city in the southwest region of the United States. Each study protocol was approved by a University Institutional Review Board.

### 2.2. Procedures

Before baseline data collection, participants and their caregivers (predominantly mothers) attended an orientation session during which research staff described the study’s objectives, procedures, and expectations. At the conclusion of this session, caregivers signed written informed consent, and participating boys and girls signed written assent. Participants were then scheduled for a 90-min baseline assessment session held either in a university classroom/gymnasium (wellness summer program) or in designated classrooms or cafeterias on school campuses (after-school program). Upon arrival at the measurement session, participants received a sealed packet containing surveys assessing (a) demographic characteristics, (b) anxiety severity, (c) sleep quality, and (d) binge-eating behaviors. Surveys were administered in a supervised setting, and research staff were available to explain instructions and clarify unfamiliar wording when needed. Participants were encouraged to ask questions during administration to ensure adequate understanding of item content. After participants completed the surveys, a research assistant led each participant to a separate room where trained staff measured participants’ height, weight, and BF% using standardized protocols.

### 2.3. Measures

#### 2.3.1. Demographic Survey

The demographic survey included 12 items that assessed demographic characteristics such as age, gender, grade, number of household family members, birthplace, as well as use of home fitness equipment and social media.

#### 2.3.2. Weight Status and Body Fat Percentage

Trained research assistants measured each participant’s height using the Seca 213 stadiometer (Seca GmbH & Co. KG, Hamburg, Germany), and body weight using a scale (Tanita SC-331S, Tanita Corporation, Tokyo, Japan). A detailed description of the assessment of weight status has already been published elsewhere [35]. Then, relying on body height and weight, research assistants computed BMI percentile values based on age- and sex-specific percentiles according to the CDC guidelines to determine weight status [40]. Participants’ body fat percentage (BF%) was also measured using foot-to-foot bioelectric impedance (Tanita SC-331S). Specifically, participants placed their bare feet on the silver footpads and stayed still until the scale estimated their BF%. The average of two BF% measurements was used for the study analysis.

#### 2.3.3. Binge Eating

The McKnight Risk Factor Survey–IV [41] was used to assess binge eating episodes along with other unhealthy weight-control strategies (not included in this study). Two items measured binge eating episodes (e.g., “In the past month, how often did you eat a lot of food in a short amount of time when it was not a meal or a holiday?” and “In the past month, how often have you kept eating and eating and felt like you could not stop?”). Questions were rated on a 5-point Likert-type scale (1 = never, 2 = a little, 3 = sometimes, 4 = a lot, and 5 = always). Per the guidelines, both items were summed to create a composite score for the binge eating subscale. Research has previously reported that the internal consistency of this measure ranges between 0.58 and 0.69 among Hispanic adolescents [42]. In the current study, the internal consistency of the binge-eating subscale was adequate, with a Cronbach’s alpha = 0.74.

#### 2.3.4. Multidimensional Anxiety Scale for Children 2nd Edition (MASC 2^TM^) 

The MASC 2^TM^ is a comprehensive measure of anxiety-related symptoms in youth aged 8 to 19 years [43]. This 50-item measure assesses a broad range of emotional, physical, cognitive, and behavioral symptoms of anxiety. The questionnaire yields raw scores and standardized T-scores for the overall degree of anxiety, six anxiety scales (i.e., separation anxiety/phobias, generalized anxiety disorder, social anxiety, obsessions and compulsions, physical symptoms, harm avoidance), and four additional subscales (i.e., humiliation/rejection, performance fears, and panic). According to the MASC 2^TM^ scoring guidelines, standardized T-scores were used for analyses in the present study. The MASC 2^TM^ is considered a valid and reliable anxiety measure, with excellent internal consistency (Cronbach’s *α* = 0.92 for the total score) [44]. In this study, the MASC 2^TM^ demonstrated excellent internal consistency (Cronbach’s *α* = 0.92).

#### 2.3.5. Pittsburgh Sleep Quality Index (PSQI) 

To assess sleep quality, we used the 19-item PSQI questionnaire [45]. The global PSQI scores range from 0 to 21, with scores ≤ 5 indicating healthy sleep quality, whereas scores > 5 indicate poor sleep quality over the past month. Previous research reported that the internal consistency of this instrument was acceptable (Cronbach’s *α* = 0.73) among adolescents [46]. In this study with early adolescents and adolescents, the internal consistency of the global PSQIs scores was good (Cronbach’s *α* = 0.82).

### 2.4. Statistical Analysis

Descriptive analyses were computed for study variables, such as frequencies and percentages for categorical variables and means and standard deviations for continuous variables. Bivariate associations among key variables were examined using Pearson correlations before regression modeling. A three-block hierarchical multiple regression analysis was conducted with binge eating frequency as the dependent variable to address the primary research questions. In Model 1, four demographic and clinical covariates were entered simultaneously: age, gender (0 = boy, 1 = girl), ethnicity (1 = Hispanic, 2 = African American/Black), and BF%. Model 2 added the two primary psychological predictors: anxiety symptom severity (MASC 2^TM^ Total Anxiety *T*-Score) and sleep quality (PSQI total score). Model 3 entered two mean-centered interaction terms, sleep quality × gender and anxiety × gender, to test whether gender moderated the relationships of the psychological predictors with binge eating. *R*^2^ change statistics were evaluated at each step to quantify the incremental variance in binge eating accounted for by each set of predictors.

Prior to computing the interaction terms, anxiety T-scores and sleep quality scores were mean centered to reduce multicollinearity between the constituent predictors and their products. Regression assumptions of normality, linearity, and homoscedasticity were evaluated through inspection of standardized residual histograms and normal P-P plots. Variance inflation factors (VIFs) for all predictors ranged from 1.01 to 1.13 across all three models, well below the conventional threshold of 10, confirming the absence of multicollinearity.

Prior to the primary analyses, cohort differences between participants enrolled in the 4-week wellness summer program and the 12-week afterschool program were examined using independent-samples of t-tests and chi-square tests. Participants in the wellness summer program were significantly older than those in the afterschool program (afterschool: *M* = 10.43, *SD* = 1.58; wellness summer: *M* = 11.57, *SD* = 1.70; *t*(313) = −5.91, *p* < 0.001); accordingly, age was retained as a covariate in all regression models. A borderline significant difference in anxiety scores was observed across cohorts [afterschool: *M* = 51.43, *SD* = 11.95; wellness summer: *M* = 48.76, *SD* = 10.51; *t*(313) = 1.97, *p* = 0.050], driven by a significant difference among boys (afterschool vs. wellness summer: *M* = 51.43 vs. *M* = 46.58; *t*(139) = 2.24, *p* = 0.027, *d* = 0.41) but not girls (*p* = 0.473). As anxiety means for both genders and cohorts fell within the normative average range (*T*-scores = 40–59), this difference was not considered clinically meaningful, and anxiety was included in regression models as planned. Sleep quality and binge eating did not differ between cohorts (all *ps* ≥ 0.583).

To characterize the pattern of missingness, excluded (*n* = 56) and included cases (*n* = 315) were compared to all key study variables. Groups did not differ significantly on age, BF%, binge eating scores, gender distribution, or program enrollment (all *ps* > 0.08). However, excluded participants reported better sleep quality (*M* = 2.08 vs. 4.04, *t*(387) = 4.70, *p* < 0.001, *d* = 0.61) and were more likely to identify as African American/Black (41.1% vs. 20.3%, *χ*^2^(1) = 10.28, *p* = 0.001). Because missingness was associated with observed variables rather than unobserved values, the pattern is consistent with a Missing At Random (MAR) rather than Missing Completely At Random (MCAR) mechanism, though MAR cannot be formally confirmed. Missing data (15.1% of cases) were therefore addressed through listwise deletion due to the limited existing data on this population, yielding an analytic sample of *N* = 315. All analyses were conducted in IBM SPSS Statistics (31.0).

## 3. Results

### 3.1. Descriptive Analyses

Of the 315 cases included in the analyses, there were slightly more girls than boys (girls: *n* = 174, 55.2%; boys: *n* = 141, 44.8%). The mean age was 10.71 years (*SD* = 1.62) for boys and 10.92 years (*SD* = 1.78) for girls. Almost 80% of the sample identified as Hispanic (Hispanic: *n* = 251, 79.7%; African American/Black: *n* = 64, 20.3%). Regarding place of birth, among the 289 participants with valid data, the majority were U.S.-born (*n* = 272, 94.1%), followed by Mexico (*n* = 9, 3.1%), Central America (*n* = 3, 1.1%), and other countries (*n* = 5, 1.7%). Regarding weight status, among the 296 participants with valid data, 4 children (1.4%) were classified as underweight, 79 (26.7%) as having a healthy weight, 54 (18.2%) as having overweight, and 159 (53.7%) as having obesity, such that 71.9% of the sample was either overweight or had obesity. Further, the mean BF% was 33.09% (*SD* = 10.58%), indicating elevated adiposity in this sample.

### 3.2. Correlations of Key Variables

As shown in Table 1, within the analytic sample (*N* = 315), the mean binge eating score was 1.86 (*SD* = 0.97, range: 1–5), indicating that, on average, children engaged in binge eating behavior to a limited extent. The mean anxiety T-score was 50.51 (*SD* = 11.53); approximately 19.4% of children (*n* = 61) scored at or above T = 60, indicating elevated anxiety symptoms, and 9.8% (*n* = 31) scored at or above T = 65, suggesting clinically significant anxiety. The mean sleep quality (PSQI) score was 4.04 (*SD* = 3.22), with higher scores reflecting poor sleep quality; approximately 26.7% of children (*n* = 84) exceeded the clinical threshold (PSQI > 5), indicating poor sleep quality. 

Bivariate correlations among study variables are presented in Table 1. Anxiety T-scores demonstrated a significant positive correlation with binge eating (*r* = 0.30, *p* < 0.001), indicating that children with higher anxiety levels reported greater frequency of engaging in binge eating behaviors. Sleep quality was similarly positively correlated with binge eating (*r* = 0.31, *p* < 0.001), such that poorer sleep, as reflected by higher PSQI scores, was associated with more frequent engagement in binge eating behaviors. Anxiety and sleep quality were also significantly correlated with each other (*r* = 0.32, *p* < 0.001), indicating that children with greater anxiety symptoms also tended to experience poorer sleep quality. In contrast, age, gender, ethnicity, and body fat percentage were not significantly associated with binge eating behaviors (see Table 1). Among the covariates, age was significantly correlated with ethnicity (*r* = 0.24, *p* < 0.01) and inversely with anxiety (*r* = −0.13, *p* < 0.05). These findings suggest that older children were more likely to be African American/Black than Hispanic and to report lower anxiety levels than younger children.

### 3.3. Hierarchical Regression Model to Predict Binge Eating

Results of the three-model hierarchical regression predicting binge eating behaviors are presented in Table 2. The covariates (age, gender, ethnicity, and BF% entered in Model 1) did not account for a statistically significant proportion of variance in binge eating, *R*^2^ = 0.009, Adj. *R*^2^ = −0.004, *F*(4, 310) = 0.726, *p* = 0.575, Δ*R*^2^ = 0.009. None of the covariates were individually significant. In Model 2, the addition of anxiety and sleep quality scores produced a large, statistically significant increment in the explained variance of binge eating behaviors, Δ*R*^2^ = 0.145, Δ*F*(2, 308) = 26.42, *p* < 0.001, bringing the total *R*^2^ to 0.154, *F*(6, 308) = 9.37, *p* < 0.001. That is, slightly more than 15% of the variance in binge eating was explained by anxiety and sleep quality, controlling for covariates. Anxiety was a significant positive predictor of binge eating (*B* = 0.020, *SE* = 0.005, *β* = 0.241, *t* = 4.30, *p* < 0.001): children with greater anxiety symptoms also reported significantly more frequent binge eating, after controlling for all covariates. Sleep quality was an equally robust positive predictor (*β* = 0.069, *SE* = 0.017, *β* = 0.230, *t* = 4.16, *p* < 0.001): poorer sleep quality was independently associated with greater binge eating, over and above anxiety and the covariates. The two predictors made nearly equal standardized contributions (*β* = 0.241 and *β* = 0.230, respectively), indicating that anxiety and sleep quality were similarly unique predictors of binge eating behavior in this sample. All four covariates remained non-significant in Model 2 (all *ps* > 0.30).

The gender moderation effects were tested in Model 3, where Sleep Quality × Gender and Anxiety × Gender terms were entered. The gender moderation effects did not produce a statistically significant increment in explained variance, Δ*R*^2^ = 0.003, Δ*F*(2, 306) = 0.46, *p* = 0.629. More specifically, neither the anxiety × gender interaction (*β* = −0.003, *SE* = 0.011, *β* = −0.014, *p* = 0.785) nor sleep quality × gender interaction (*β* = −0.028, *SE* = 0.031, *β* = −0.048, *p* = 0.361) reached statistical significance. The adjusted *R*^2^ decreased marginally from 0.138 in Model 2 to 0.135 in Model 3, further confirming that the interaction terms did not improve model fit. The main effects of anxiety (*β* = 0.241, *p* < 0.001) and sleep quality (*β* = 0.229, *p* < 0.001) remained stable and significant in the full model, demonstrating the robustness of these associations regardless of the gender interaction terms. Taken together, these findings indicate that gender did not moderate the relationships of either anxiety or sleep quality with binge eating behaviors in this sample.

## 4. Discussion

The present study examined the associations among anxiety, sleep quality, and binge-eating behaviors in a sample of Hispanic and African American/Black early adolescents with elevated risk for overweight and obesity, while also testing whether these relationships were moderated by gender. Consistent with our hypotheses, higher levels of anxiety and poorer sleep quality were both significantly associated with greater engagement in binge-eating behaviors. Contrary to expectations, however, gender did not moderate these relationships. The present findings are interpreted through the integrated theoretical lens of affect regulation theory, psychosocial stress theory, and restraint theory. Collectively, these frameworks propose that when psychological and behavioral stressors, such as anxiety and sleep disruption, exceed an individual’s self-regulatory capacity, binge eating can emerge as a maladaptive coping response. Together, these findings underscore the importance of psychosocial and behavioral factors in understanding binge-eating risk among racially and ethnically diverse youth and provide support for this integrated theoretical model.

The finding that anxiety was positively associated with binge eating aligns with a growing body of literature indicating that emotional distress plays a central role in the development of disordered eating behaviors during adolescence. The experience of elevated anxiety among youth has been positively associated with binge eating, potentially reflecting the use of maladaptive coping strategies to regulate negative affect [31]. Specifically, affect regulation theory posits that binge eating functions as an escape from aversive self-awareness: when anxiety triggers overwhelming negative affect, individuals lacking effective emotional regulation skills may engage in binge eating behaviors to temporarily reduce psychological discomfort [23,24]. In this way, binge eating is not simply correlated with anxiety but is theorized to be functionally maintained by it. This interpretation is consistent with affect regulation theory, psychosocial stress theory, and restraint theory, which posit that psychological and behavioral factors, such as anxiety, sleep quality, and emotional dysregulation, interact to shape health behaviors. Given that nearly one in five participants in the present study reported elevated anxiety symptoms, these findings highlight anxiety as a clinically meaningful target for early intervention.

Similarly, sleep quality emerged as an independent and comparably strong predictor of binge eating. That is, adolescents in this sample who reported poorer sleep quality engaged in more frequent binge eating behaviors, even after accounting for anxiety and demographic factors. This finding is consistent with prior research suggesting that insufficient or disrupted sleep is associated with increased risk for binge eating and may contribute to dysregulation of appetite-related hormones and impairment in self-regulation and impulse control [33,34]. From a restraint theory perspective, sleep-related impairments in self-regulation may lower the threshold for engaging in loss-of-control eating, particularly among youth already navigating weight-related pressures [25,26]. Furthermore, psychosocial stress theory suggests that the chronic stressors disproportionately experienced by Hispanic and African American/Black youth, including socioeconomic adversity, weight stigma, and discrimination, may simultaneously elevate anxiety and disrupt sleep, creating a compounding pathway toward binge-eating risk. Importantly, the comparable magnitude of the standardized regression coefficients for anxiety and sleep suggests that both factors may play equally important roles in shaping binge-eating behaviors in early adolescence. The moderate correlation observed between anxiety and sleep further supports the notion of an interconnected, bidirectional relationship among these processes. This potential bidirectional relationship is further supported by evidence that sleep disturbances heighten anxiety and impair emotional regulation [35], and that these associations persist across racially and ethnically diverse populations, with some studies reporting comparable or even elevated prevalence of disordered eating among minority youth [15,16,34].

Contrary to our second hypothesis, gender did not moderate the relationships between anxiety, sleep quality, and binge eating. This finding suggests that the associations of anxiety and sleep with binge-eating behaviors may operate similarly for boys and girls in this sample. From a psychosocial stress theory perspective, this pattern may reflect the relatively homogeneous stress exposure within this sample; that is, all participants belonged to minority groups with elevated obesity risk and shared similar psychosocial burdens, potentially attenuating gender-related differences that might otherwise emerge in less uniformly stressed populations. Further, boys and girls did not significantly differ (not presented) in binge eating behavior scores, although prior research has more consistently documented higher rates of disordered eating among girls [9]. Emerging evidence, however, indicates that boys are also vulnerable to disordered eating behaviors, particularly within racially and ethnically diverse or higher-risk populations [17]. The lack of difference between boys and girls may reflect the relatively young age of the sample, as gender differences in disordered eating behaviors may become more pronounced later in adolescence. Indeed, the youth in this sample reported, on average, engaging in binge eating behaviors slightly less than “a little” on the response scale (mean = 1.86). Alternatively, it may indicate that psychosocial stressors such as anxiety and sleep disturbance exert a broadly similar influence across genders in early developmental stages.

Interestingly, demographic and weight-related variables, including age, ethnicity, and BF%, were not significantly associated with binge eating in this sample. While this may initially appear inconsistent with prior research linking obesity and sociocultural factors to disordered eating [5,15,18], it is important to note that the present sample was relatively homogeneous with respect to weight status, with approximately 70% of participants classified as having either overweight or obesity. This restricted variability may have attenuated the ability to detect associations between demographic and weight-related variables and binge eating. Instead, the findings suggest that within populations of preadolescents at high risk of obesity, psychological and behavioral factors may be more proximal predictors of binge-eating behaviors than demographic characteristics alone.

These findings have several important implications for both clinical practice and prevention efforts. First, they highlight the need for early screening and intervention strategies that address anxiety symptoms and sleep disturbances among youth at risk for obesity and disordered eating. Integrating mental health and sleep assessments into pediatric and school-based health programs may facilitate early identification of youth at risk for binge eating. Second, the findings support the value of integrated interventions that simultaneously target emotional regulation, stress management, and sleep hygiene. Programs incorporating cognitive-behavioral strategies have demonstrated effectiveness in reducing binge-eating behaviors [18]. Grounded in affect regulation theory, such programs directly target the emotional dysregulation mechanism identified as central to binge-eating maintenance and may therefore be theoretically well suited to this population. Additionally, emerging research highlights the potential role of mindfulness-based approaches [47] and the role of sleep on binge eating behaviors [33,34], suggesting that interventions targeting these factors may also be beneficial. Given the disproportionate burden of obesity and related health disparities among Hispanic and African American/Black youth, culturally responsive interventions that consider contextual factors such as socioeconomic stress, food environments, and cultural norms are critical. The absence of gender moderation effects suggests that prevention and intervention programs should be inclusive of both boys and girls rather than primarily targeting female populations, as has historically been the case in eating disorder research and treatment.

Several limitations should be considered when interpreting these findings. First, the cross-sectional design precludes conclusions about causality or temporal ordering among anxiety, sleep quality, and binge eating. Longitudinal studies are needed to determine whether anxiety and sleep disturbances prospectively predict the onset or escalation of binge-eating behaviors. Second, all psychological and behavioral measures were based on self-report, which may be subject to recall bias or social desirability effects. Although validated instruments were used, incorporating multi-method assessments (e.g., clinical interviews, actigraphy for sleep) would strengthen future research. Third, the measurement of binge eating relied on two self-report items assessing frequency of behaviors, which may not fully capture the complexity or clinical severity of binge-eating episodes. However, because the intention was to capture information about maladaptive binge eating behaviors at the subclinical level rather than diagnose BED, the two self-report items provide insights into associated correlates in this sample. Nevertheless, future research may wish to include more comprehensive assessments to better characterize loss-of-control eating and binge eating severity such, as the Binge Eating Scale [48], Eating Disorder Examination Questionnaire [49], or Loss of Control over Eating Scale [50].

Fourth, the limitation of the appropriateness and validity of the PSQI measure for children should be acknowledged. In particular, the psychometric properties of the PSQI may be less established for younger children, particularly those aged 9–10 years. We also acknowledge that some instruments may not fully capture culturally specific experiences or perspectives within the populations represented in our sample. As a result, future studies should include instruments that assess culturally specific experiences (e.g., discrimination or immigration harassment). Fifth, the extended recruitment period (2016–2019) may introduce temporal biases into the sample, with changes over time in participant characteristics, external conditions, or program delivery, which may have influenced the sample and outcomes. These factors should be considered when interpreting the findings and assessing their external validity. Likewise, given that the participants were recruited exclusively from wellness and after-school mindful programs, this may have introduced selection bias, as children and adolescents who participate in these programs are likely to be more concerned about their weight, motivated, and receptive to wellness-related interventions than the general population. Thus, findings should be interpreted with caution, as they may not fully represent all children and adolescents.

In addition, although systematic comparisons of participants across the two cohorts (wellness summer and afterschool programs) revealed that cohort differences were largely limited to age and, among boys only, anxiety scores within the normative range, the possibility that unmeasured between-cohort differences (e.g., program content, setting, or temporal factors) influenced the findings cannot be fully excluded. Finally, missing data (15.1% of cases) were handled via listwise deletion. Although comparisons of included and excluded participants were consistent with a Missing at Random (MAR) mechanism, listwise deletion under MAR may introduce some bias relative to more robust approaches such as multiple imputation (MI) or full-information maximum likelihood (FIML) estimation. Future research with this population should employ these methods to reduce potential bias.

Future research should build on these findings by employing longitudinal designs to examine developmental trajectories and causal pathways linking anxiety, sleep disturbances, and binge eating. Additionally, examining other relevant psychosocial factors—such as body image dissatisfaction, depression, stress exposure, food insecurity, and experiences of discrimination—may provide a more comprehensive understanding of risk pathways, particularly among racially and ethnically diverse youth. Further investigation into potential protective factors, such as family support, coping skills, cultural identity, and body image, may also help inform strength-based interventions. Finally, research should continue to explore subgroup differences within Hispanic and African American/Black female and male populations to better understand within-group heterogeneity [51].

## 5. Conclusions

In summary, the present study demonstrates that anxiety and poor sleep quality are significant and independent predictors of binge-eating behaviors among Hispanic and African American early adolescents at risk for overweight and obesity. These findings support an affect regulation, psychosocial stress, and restraint framework in which psychological and behavioral factors play a central role in the development of disordered eating. Early, integrated interventions targeting anxiety and sleep may be critical for reducing binge-eating risk and addressing broader health disparities in this vulnerable population.

## Figures and Tables

**Table 1:** Mean, Standard Deviation, and Correlations Among Key Variables (*N* = 315).

Variable	M	SD	1	2	3	4	5	6	7
1. Binge Eating	1.86	0.97	—						
2. Age	10.83	1.71	0.04	—					
3. Gender	0.55	0.50	−0.03	0.06	—				
4. Ethnicity	1.20	0.40	0.06	0.24 **	0.04	—			
5. Body Fat Percentage	33.09	10.58	−0.07	0.01	0.03	−0.02	—		
6. Anxiety T-Score	50.51	11.53	0.30 ***	−0.13 *	0.04	−0.05	−0.03	—	
7. Sleep Quality	4.04	3.22	0.31 ***	0.04	0.01	0.01	−0.02	0.32 ***	—

Note. Gender coded: 0 = Boy; 1 = Girl. Ethnicity coded: 1 = Hispanic; 2 = African American/Black. Body fat percentage estimated via bioelectrical impedance analysis (BIA). Sleep Quality = global score (higher = poorer sleep). Anxiety = MASC-2™ Total Anxiety T-Score * *p* < 0.05, ** *p* < 0.01, *** *p* < 0.001.

**Table 2:** Hierarchical Regression Analysis Predicting Binge Eating (*N* = 315).

Variables	B	SE	*β*	t	R^2^	Adj. R^2^
Model 1					0.009	−0.004
Age	0.019	0.033	0.034	0.584		
Gender	−0.065	0.110	−0.033	−0.588		
Ethnicity	0.114	0.139	0.048	0.817		
Body Fat Percentage	−0.006	0.005	−0.064	−1.128		
Model 2					0.154 ***	0.138
Age	0.032	0.031	0.056	1.029		
Gender	−0.092	0.102	−0.047	−0.897		
Ethnicity	0.131	0.129	0.055	1.016		
Body Fat Percentage	−0.005	0.005	−0.051	−0.972		
Anxiety T-Score	0.020	0.005	0.241 ***	4.296		
Sleep Quality	0.069	0.017	0.230 ***	4.155		
Model 3					0.157	0.135
Age	0.032	0.031	0.057	1.037		
Gender	−0.095	0.102	−0.049	−0.925		
Ethnicity	0.124	0.130	0.052	0.952		
Body Fat percentage	−0.005	0.005	−0.051	−0.977		
Anxiety T-Score	0.020	0.005	0.241 ***	4.291		
Sleep Quality	0.069	0.017	0.229 ***	4.119		
Sleep × Gender	−0.028	0.031	−0.048	−0.915		
Anxiety × Gender	−0.003	0.011	−0.014	−0.274		

Note. Gender coded: 0 = Boy; 1 = Girl. Ethnicity coded: 1 = Hispanic; 2 = African American/Black. Body fat percentage estimated via bioelectrical impedance analysis (BIA). Sleep Quality = global score (higher = poorer sleep). Anxiety = MASC-2 Total Anxiety *T*-Score. Interaction terms computed from mean-centered predictors. VIFs ranged from 1.01 to 1.13 across all models. *** *p* < 0.001.

## Data Availability

The data are not publicly available due to concerns regarding privacy.
